# Blue Electroluminescent Al_2_O_3_/Tm_2_O_3_ Nanolaminate Films Fabricated by Atomic Layer Deposition on Silicon

**DOI:** 10.3390/nano9030413

**Published:** 2019-03-11

**Authors:** Yao Liu, Zhongtao Ouyang, Li Yang, Yang Yang, Jiaming Sun

**Affiliations:** School of Materials Science and Engineering, Tianjin Key Lab for Rare Earth Materials and Applications, Nankai University, Tianjin 300350, China; 15100299616@163.com (Y.L.); zhongtoe@163.com (Z.O.); materialyang@126.com (L.Y.)

**Keywords:** electroluminescence, nanolaminate, Al_2_O_3_, Tm_2_O_3_, atomic layer deposition

## Abstract

Realization of a silicon-based light source is of significant importance for the future development of optoelectronics and telecommunications. Here, nanolaminate Al_2_O_3_/Tm_2_O_3_ films are fabricated on silicon utilizing atomic layer deposition, and intense blue electroluminescence (EL) from Tm^3+^ ions is achieved in the metal-oxide-semiconductor structured luminescent devices based on them. Precise control of the nanolaminates enables the study on the influence of the Tm dopant layers and the distance between every Tm_2_O_3_ layer on the EL performance. The 456 nm blue EL from Tm^3+^ ions shows a maximum power density of 0.15 mW/cm^2^. The EL intensities and decay lifetime decrease with excessive Tm dopant cycles due to the reduction of optically active Tm^3+^ ions. Cross-relaxation among adjacent Tm_2_O_3_ dopant layers reduces the blue EL intensity and the decay lifetime, which strongly depends on the Al_2_O_3_ sublayer thickness, with a critical value of ~3 nm. The EL is attributed to the impact excitation of the Tm^3+^ ions by hot electrons in Al_2_O_3_ matrix via Poole–Frenkel mechanism.

## 1. Introduction

Traditional electronic integrated circuits have been facing with a bottleneck in terms of power consumption, speed, and signal crosstalk as the communication frequency and bandwidth rise to a higher level. One possible solution is the optoelectronic integration which realizes photonic technologies on silicon chips [1,2,3,4]. However, applicable Si-based light sources have been unsolved for a long time. Rare earth (RE) ions are generally efficient luminescence centers in various matrixes. Nowadays diverse RE-doped insulating materials have been developed for the applications in solid state lasers and phosphors [5,6,7,8,9]. However, it has been widely known that the mismatch in the coordination structure and atomic size of silicon (tetrahedron) and RE ions (octahedron) limit the desired spectroscopic performance due to the clustering of RE ions in the Si host [10,11]. Aiming for the realization of compact Si-based optoelectronics, electroluminescence (EL) from RE^3+^ ions has been extensively reported in many compounds, such as SiN_x_, TiO_2_, and ZnO [12,13,14,15]. However, the efficiencies of the devices based on the aforementioned materials are far from practical utilization. One of the limitations is the large leakage current. RE-implanted SiO_2_ MOS-structured light-emitting devices (MOSLEDs) have attracted much attention due to their notable EL efficiency and silicon compatibility [16,17]. In comparison, similar devices based on Al_2_O_3_ nanofilms present much lower working voltage, and comparable efficiency in our previous study, while their EL performance needs more exploration [18,19]. Blue emission, which has the highest photon energy (2.6–2.7 eV) of the three primary colors, is of great importance in display and lighting. Tm^3+^ ions have present efficient blue emissions in various matrixes including ZnS, ZnO, fluorophosphate, and many other oxides and fluorides [20,21,22,23]. The reported achievements are mostly focused on photoluminescence (PL), by virtue of upconversion to convert infrared photons to blue emission [20,24]. For practical application, electrically excited devices are urgently needed. Whether high-energy blue photons can be generated in this prototype device is still unknown. Using Tm-doped Al_2_O_3_ might exploit the merits of both oxides to realize efficient blue EL from Tm^3+^ ions.

Atomic layer deposition (ALD) is a monatomic vapor deposition technique achieved by alternating saturated gas–surface reactions, based on which the film can be deposited in a self-limited growth mode and exhibits superior homogeneity and excellent uniformity [25,26,27,28,29]. This technique supplies a convenient way to devise nanolaminates with optimal performance. In this work, we fabricate nanolaminate Al_2_O_3_/Tm_2_O_3_ films which function as blue EL layers in the Si-based MOSLEDs. The EL intensity and decay lifetime are compared by changing the Al_2_O_3_ or Tm_2_O_3_ sublayer cycles. The influence of the Tm clustering and interaction concerning the Al_2_O_3_ or Tm_2_O_3_ cycles are explored respectively. The 456 nm blue EL from Tm^3+^ ions shows a maximum power density of 0.15 mW/cm^2^. The device characteristics are in good consistence with the previous reports on the excitation mechanism and the critical interlayer thickness for the cross-relaxation among adjacent dopant layers.

## 2. Materials and Methods

The nanolaminate Al_2_O_3_/Tm_2_O_3_ films were grown on <100>-oriented phosphorous–doped silicon (n-Si) substrates with the resistivity of 2–5 Ω·cm and a thickness of 500 μm (CETC-46 Ltd., Tianjin, China), which were cleaned through the standard RCA process before growth. The ALD equipment was a 4-inch chamber system (Nano Tech Savannah 100, Cambridge, MA, USA). Trimethylaluminum [TMA, Al(CH_3_)_3_, 99.999+%] and Tm(THD)_3_ (THD = 2,2,6,6-teramethyl-3,5 heptanedionate, 99.9%, Strem Chemicals, Inc., Newburyport, MA, USA) were used as the metal precursors for Al_2_O_3_ and Tm_2_O_3_, while ozone was used as the oxidant. N_2_ was used as the carrier and purge gas with a flow rate of 20 sccm. During the growth, the pulse time of TMA, Tm(THD)_3_, and ozone was 0.015 s, 2 s, and 1.8 s, respectively. The TMA was maintained at room temperature while the Tm precursor was heated at 170 °C. The pipelines and the substrates were maintained at 190 °C and 325 °C. The growth rates for the Tm_2_O_3_ and Al_2_O_3_ films were 0.216 Å/cycle and 0.79 Å/cycle, respectively.

In order to investigate the luminescent characteristics of nanolaminate Al_2_O_3_/Tm_2_O_3_ films, a series of devices concerning the Tm_2_O_3_ dopant cycles and the Al_2_O_3_ interlayer cycles were fabricated as shown in Table 1. The total cycle numbers were adjusted correspondingly to obtain the luminescent films with a thickness of ~50 nm. The thickness of the film was measured by an homemade ellipsometer with a 632.8 nm He-Ne laser at an incident angle of 69.8°. As the thickness variation from the designed value for the nanolaminates are quite small (less than 3%), the nominal Tm concentrations are used to quantify the doping levels. All Al_2_O_3_/Tm_2_O_3_ films were subsequently annealed at 800 °C in N_2_ atmosphere for 1 h to reduce defects and activate Tm^3+^ luminescence. Then, 120 nm TiO_2_/Al_2_O_3_ nanolaminate films consisting of 2 nm Al_2_O_3_ and 8 nm TiO_2_ sublayers were grown by ALD on Al_2_O_3_/Tm_2_O_3_ films as the protective layers. Afterwards, ~100 nm ZnO:Al_2_O_3_ films were grown by ALD as the transparent conductive electrodes, which were lithographically patterned into 0.5 mm circular dots. Finally, 100 nm Al electrodes were deposited on the back side of the Si substrates by thermal evaporation, and annealed afterwards in vacuum at 250 °C for 0.5 h to realize ohmic contact.

The PL spectra from the luminescent nanolaminates were excited by a 355 nm laser. For EL and Current–Voltage (I–V) measurements, the devices were activated by means of a Keithley 2410 SourceMeter unit (Keithley Instruments Inc., Cleveland, OH, USA), with the negative voltage connecting to n-Si substrates. The PL and EL signals were detected by a monochromator (Zolix λ500, Zolix Instruments Co., Ltd, Beijing, China) and a Si photomultiplier connected to a Keithley 2010 multimeter (Keithley Instruments Inc., Cleveland, OH, USA). Photographic images were collected by a digital camera through a 20-fold objective microscope. Time-resolved photoluminescence (TRPL) was measured by a SR430 multi-channel scaler (Stanford Research Systems Inc., Sunnyvale, CA, USA) with a 355 nm laser working in the pulse mode. The decay lifetime of the EL emission was measured by the SR430 multichannel scaler, excited by a high-voltage amplifier equipped with a digital function signal generator (DG5072, RIGOL Technology Co., Ltd, Beijing, China). All the above measurements were performed at room temperature.

## 3. Results and Discussion

**Figure 1:**
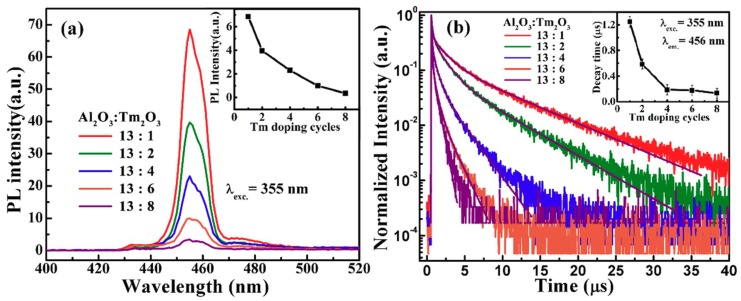
The (**a**) photoluminescence (PL) and (**b**) time-resolved photoluminescence (TRPL) spectra from the nanolaminate Al_2_O_3_/Tm_2_O_3_ films with different Tm dopant cycles excited by a 355 nm laser. The insets present the tendency of these PL intensities and PL decay lifetime with the Tm dopant cycles.

The Tm_2_O_3_ films deposited by ALD can be crystalized into Tm_2_O_3_ phase even without annealing, while the Al_2_O_3_ films are amorphous after annealing at 800 °C. However, the nanolaminate Al_2_O_3_/Tm_2_O_3_ film with the highest Tm content (AOT-8) is amorphous after annealing at 800 °C, therefore the nanolaminate structure restricts the grain growth of the dopant Tm_2_O_3_ layers. Figure 1a shows the PL spectra from the nanolaminate Al_2_O_3_/Tm_2_O_3_ films. The PL peaks at 456 nm are attributed to the transition of ^1^D_2_→^3^F_4_ in Tm^3+^ ions [20,21,22]. The inset of Figure 1a presents the comparison of the PL intensities of all samples, which decrease with the Tm_2_O_3_ dopant layers. Due to the common cluttering characteristics of RE ions, with the increase of Tm content, the number of activated Tm^3+^ ions decreases and the cross relaxation between Tm^3+^ ions further reduce the radiative transitions [30,31]. For TRPL results shown in Figure 1b, the decay lifetime of these PL emissions from Tm^3+^ ions also decreases with the Tm content, which coincides with the PL intensities. The inset gives the fitting values of the PL decay lifetime, which are in the range of 0.13–1.25 μs. The PL decay lifetime decreases rapidly as the Tm dopant layers rise to 4. The cross relaxation and concentration quenching contribute to the nonradiative recombination and decrease the luminescence lifetime.

**Figure 2:**
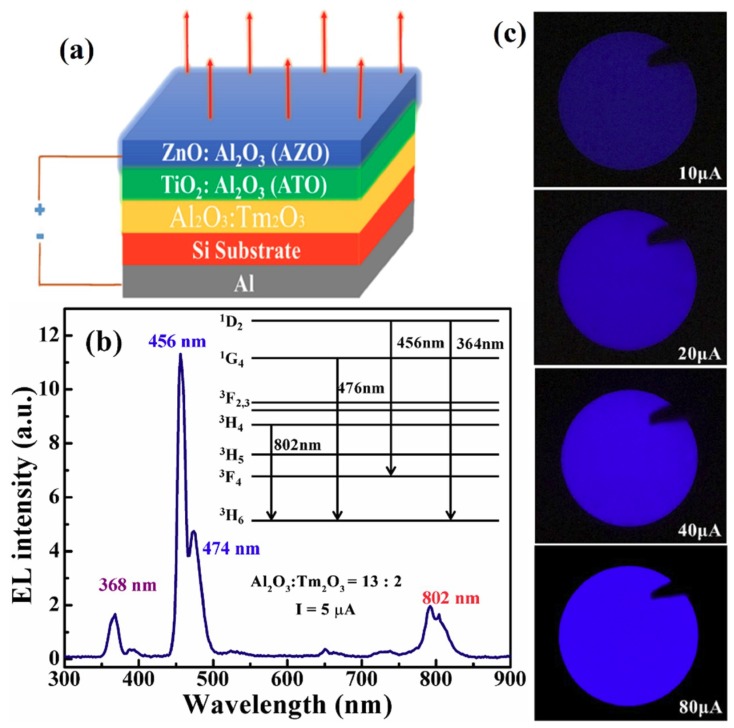
(**a**) The schematic for the luminescent devices based on the nanolaminate Al_2_O_3_/Tm_2_O_3_ films. (**b**) The EL spectrum from the device in which the Al_2_O_3_/Tm_2_O_3_ subcycle ratio is 13:2 (AOT-2), the inset shows the radiative transitions in the Tm^3+^ ions resulting in the EL emissions. (**c**) The images taken by a digital camera from this AOT-2 MOS-structured light-emitting device (MOSLED) at different injection currents.

The schematic for the multilayered devices is shown in Figure 2a. The EL spectrum from the MOSLED based on the Al_2_O_3_/Tm_2_O_3_ nanolaminate with 2 cycles of Tm dopant (AOT-2) is presented in Figure 2b. The EL emissions mainly exhibit several peaks at the wavelengths of 368, 456, 474, and 802 nm, which originate from the radiative transitions from the ^1^D_2_, ^3^F_4_, ^1^G_4_, and ^3^H_4_ excited states to the ^3^H_6_ ground state in Tm^3+^ ions, respectively, as sketched in the inset of Figure 2b [21,22,23]. It is noteworthy that the EL emissions at 456 nm and 474 nm are dominating and the blue light is easily seen by naked eyes, as shown in Figure 2c. These images were taken by a digital camera from this AOT-2 MOSLED at different injection currents. The blue EL emission gradually brightens with the increase of the injection current from 10 µA to 80 µA.

**Figure 3:**
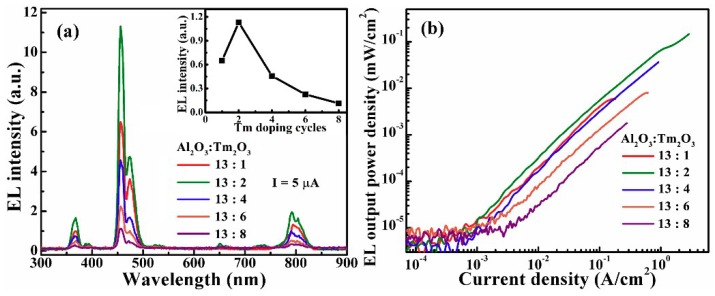
(**a**) EL spectra from the MOSLEDs based on the Al_2_O_3_/Tm_2_O_3_ films with different Tm dopant cycles at an injection current of 5 µA, the inset shows the tendency of this EL intensity with the Tm dopant layers. (**b**) The dependence of the 456 nm EL power density on the injection current density for the Al_2_O_3_/Tm_2_O_3_ MOSLEDs with different Tm dopant cycles.

Figure 3a shows EL spectra from the MOSLEDs based on the Al_2_O_3_/Tm_2_O_3_ films with different Tm dopant cycles at an injection current of 5 µA. The concentrations of Tm dopant are from 0.69% to 4.95%, respectively. The spectra exhibit four peaks at 368, 458, 474, and 802 nm as mentioned above. The inset shows that the 456 nm blue EL intensity increases with the Tm dopant cycles up to 2 and then decreases due to concentration quenching. The EL presents higher tolerance for Tm clustering than the PL performance. The dependence of the 456 nm EL power density on the injection current density are shown in Figure 3b. Generally, the EL intensity presents a linear relationship with the injection current density. A power density up to 0.15 mW/cm^2^ was obtained from the optimal MOSLED at a current density of 2.87 A/cm^2^. Initially, the EL output power density increases as the Tm dopant cycles increases to 2, due to the increase of the excitable Tm^3+^ ions. The further decline of the power density with the Tm dopant cycle is attributed to the clustering and cross relaxation which reduce the number of excited Tm^3+^ ions [30,31]. The efficiency and output power are lower than the previously reported devices based on the Tb and Yb doped Al_2_O_3_ nanolaminates [18,19]. As the energy of the blue photon is higher than that of the green EL from Tb^3+^ ions and the near-infrared one from Yb^3+^ ions, the excitation possibility of the radiative transitions within Tm^3+^ ions should be lower which leads to the limited efficiency and output power. In addition, the visible EL from the RE-doped SiO_2_ is stronger than the devices in this work [32]. The higher working voltage needed for luminescence in SiO_2_ evidences the necessity of high electrical field for excitation of the photon with higher energy, which is adverse to practical application. However, this EL output power density is superior to the EL devices based on the RE-doped ZnO as the leakage current is greatly restricted comparatively [13].

**Figure 4:**
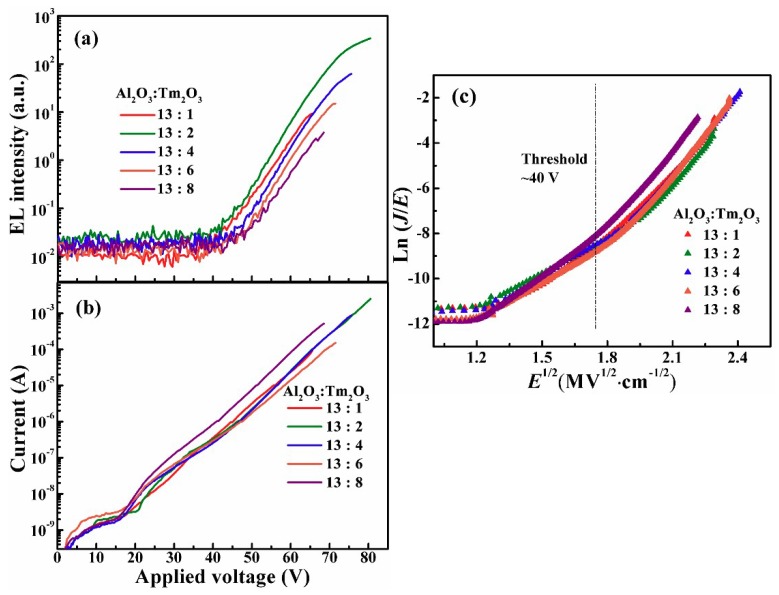
(**a**,**b**) The dependence of blue (456 nm) EL intensities, together with the injection current, on the applied voltages for the nanolaminate MOSLEDs based on different Al_2_O_3_/Tm_2_O_3_ films. (**c**) The plot of ln(*J*/*E*) versus *E*^1/2^ (Poole–Frenkel conduction mode) for these MOSLEDs.

Figure 4a,b shows the dependence of blue (456 nm) EL intensities, together with the injection current, on the applied voltages for the nanolaminate MOSLEDs based on different Al_2_O_3_/Tm_2_O_3_ films. All devices exhibit a typical I–V characteristic of the MOS structure, i.e., the current starts with a low background one under the low electric field, then exponentially increases with the voltage [16,17,18,19]. The difference on the leakage currents mainly depends on the process of device procedures, coming from the electrons hopping through the defects within the matrix. At this stage, no hot electrons are generated in the Al_2_O_3_/Tm_2_O_3_ conduction band with no EL emissions. Afterwards, the injection current increases exponentially with the applied voltage and the conduction mechanism is dominated by the Poole–Franked (P–F) mode until the device breakdown [18,19]. In the P–F conduction mode the plot of the ln(*J*/*E*) versus *E*^1/2^ features a linear relationship (*J* is the current density and *E* is the electric field). As shown in Figure 4c, for all Al_2_O_3_/Tm_2_O_3_ MOSLEDs the P–F plots work in the EL-enabling voltages, with the threshold voltage of around 40 V (~3 MV/cm). The slopes of the linear plots of the P–F injections are similar while the little difference is caused by the slight variation of the injection current as mentioned above. Therefore, for the EL excitation, electrons are firstly injected into the conduction band of Al_2_O_3_ by trap-assisted tunneling and accelerated to gain energy under high electric field. These hot electrons excite the doped Tm^3+^ ions from the ground state to higher levels by inelastic collision. After the nonradiative relaxation, the radiative transitions in the Tm^3+^ ions from the excited state to ground state generate the characteristic EL emissions [20,21,22].

**Figure 5:**
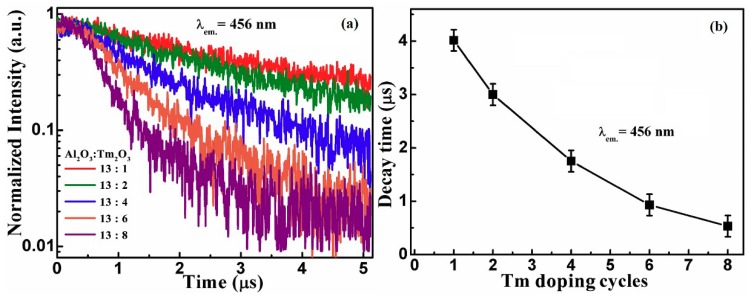
(**a**) The EL decay lifetime of the 456 nm EL from different nanolaminate Al_2_O_3_/Tm_2_O_3_ MOSLEDs and (**b**) the tendency of the EL decay lifetime with the Tm dopant cycles.

The EL decay lifetime of the 456 nm EL from different nanolaminate Al_2_O_3_/Tm_2_O_3_ MOSLEDs is measured under pulse excitation mode. The decay curves are shown in Figure 5a, which are close to the single exponential decay function. The decay lifetime decreases from 4.02 µs to 0.53 µs with the increase of Tm dopant cycles, as shown in Figure 5b. These values of EL decay lifetime are several times larger than that of PL decay lifetime shown in Figure 1b, and keep decreasing with the Tm doping concentration, which comes from the cross relaxation and concentration quenching caused by the excess Tm^3+^ ions. These phenomena again mean that the tolerance on the concentration quenching in EL performance is higher than that in PL.

**Figure 6:**
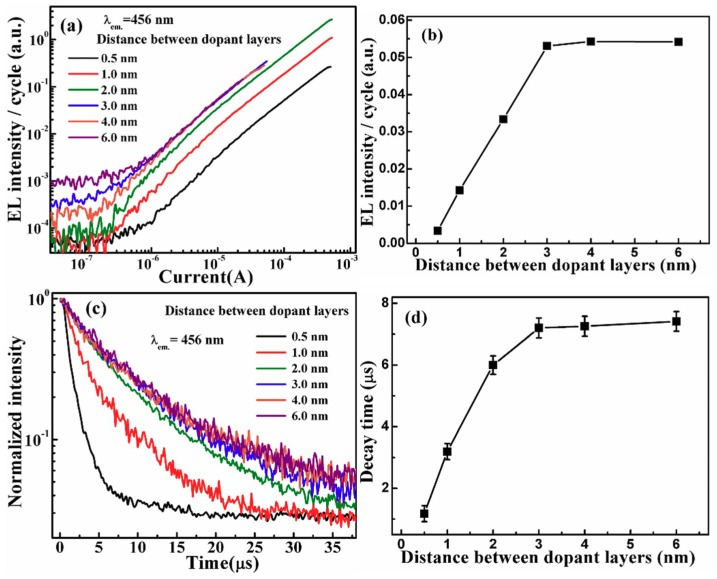
(**a**) The dependence of the blue EL intensity on the injection current from the Al_2_O_3_/Tm_2_O_3_ MOSLEDs with different Al_2_O_3_ sublayer thicknesses. Here, the EL intensities are divided by the cycle numbers to present the emissions from every Tm dopant cycle. (**b**) The relation of blue EL intensity with the Al_2_O_3_ sublayer thicknesses. (**c**) The EL decay lifetime for the Al_2_O_3_/Tm_2_O_3_ MOSLEDs and (**d**) the relation of lifetime to the Al_2_O_3_ sublayer thicknesses.

In the RE-doped Al_2_O_3_ MOSLEDs, the Al_2_O_3_ sublayer thickness affects the cross relaxation between excited RE ions, and the acceleration distance for injected electrons. In order to investigate the effect of the distance between Tm_2_O_3_ dopant layers, a series of MOSLEDs were fabricated in which the Al_2_O_3_ sublayer thickness varied from 0.5 nm to 6 nm while the Tm dopant cycles was fixed at 2. Figure 6a shows the dependence of the blue EL intensity on the injection current. Here, the EL intensities are divided by the cycle numbers to present the emissions from every Tm dopant cycle. With the increase of the thickness of Al_2_O_3_ sublayer, the contribution of a single Tm dopant cycle to the EL intensity firstly increases and then saturates as the Al_2_O_3_ interlayer thickness reaches 3 nm. Figure 6b presents the tendency. This phenomenon has been observed in our previous reports with a similar value, concerning the nonradiative interaction among excited RE^3+^ ions and the acceleration distance for the injected electrons [18,19]. Therefore, it is a common characteristic for the luminescent RE^3+^ ions in an Al_2_O_3_ matrix that the distance for the presence of nonradiative interaction and adequate electron acceleration is around 3 nm.

Furthermore, the decay lifetimes for these MOSLEDs are shown in Figure 6c, whose correlation with the Al_2_O_3_ interlayer thickness is summarized in Figure 6d. Similar to the EL intensity, the decay lifetime increases from 1.18 to 7.41 μs with the Al_2_O_3_ interlayer thickness increasing from 0.5 nm to 3 nm, and saturates at higher distances. The reduction of the decay lifetime at higher Tm doping concentrations is still ascribed to the increase of nonradiative cross relaxations between the two closely located Tm^3+^ dopant layers as mentioned above, with the similar critical Al_2_O_3_ interlayer thickness of 3 nm [19,33]. Considering the total EL intensities, the optimal Al_2_O_3_ interlayer thickness in these MOSLEDs is 2 nm. It should be noted that there is little difference between the total EL emission from nanolaminate Al_2_O_3_/Tm_2_O_3_ MOSLEDs with 1 nm and 2 nm Al_2_O_3_ interlayers. The effect of more dopant ions is offset by the relative lowered excitation efficiency. This optimal doping concentration is also consistent with previous reports (around 1 at%) on the RE doped luminescent materials [18,33].

The blue EL intensities (output powers) from our prototype devices are quite low and incapable of practical application. This work confirms the potential to realize blue EL from Al_2_O_3_/Tm_2_O_3_ nanolaminates by ALD. Moreover, the devices are fabricated entirely by ALD, which is characterized by the precise control of the film deposition over large substrates, and the compatibility with Si-based CMOS technology. Therefore, MOSLEDs based on Al_2_O_3_/Tm_2_O_3_ nanolaminates can be easily expanded for mass-production. The challenging deficiencies are the low EL efficiency and output power, the high working voltage, and the limited injection current. Further optimization can be achieved by adopting a thicker Al_2_O_3_/Tm_2_O_3_ luminescent layer with more optimal dopant structure and a less resistant protective layer with higher dielectric constant, to obtain a higher emission intensity.

## 4. Conclusions

Blue EL is demonstrated from nanolaminate Al_2_O_3_/Tm_2_O_3_ MOSLEDs fabricated by ALD. The emission at 456 nm from Tm^3+^ ions exhibits a power density of 0.15 mW/cm^2^. The decrease of the EL intensity and decay lifetime due to the clustering and cross-relaxation of the Tm^3+^ ions is observed by adjusting the Tm_2_O_3_ dopant cycles. The decay lifetime for the Tm^3+^ ions under optical excitation is in the range of 0.13–1.25 μs while under electrical excitation, the decay lifetime increases to 1.13–4.02 μs. The EL is attributed to the impact excitation of the Tm^3+^ ions by hot electrons in the Al_2_O_3_ matrix via the P–F mechanism. Consistent with the previous results, a critical Al_2_O_3_ interlayer thickness of ~3 nm for the nonradiative interaction among excited Tm^3+^ ions and the acceleration distance of the injected electrons works. This work could contribute to the development of Si-compatible RE-doped light sources by modifying the dopant structure in the nanolaminates to achieve efficient emissions.

## Figures and Tables

**Table 1:** The corresponding experimental parameters for clarity.

Sample Label	Tm^3+^ (at%)	Al_2_O_3_:Tm_2_O_3_ Cycle Number
AOT-1	0.69	13:1
AOT-2	1.37	13:2
AOT-4	2.64	13:4
AOT-6	3.83	13:6
AOT-8	4.95	13:8
AOT-d05	2.46	7:2
AOT-d1	1.37	13:2
AOT-d2	0.69	26:2
AOT-d3	0.45	40:2
AOT-d4	0.35	52:2
AOT-d6	0.23	78:2

## References

[1] Gan X., Shiue R.J., Gao Y., Meric I., Heinz T.F., Shepard K., Hone J., Assefa S., Englund D. (2013). Chip-integrated ultrafast graphene photodetector with high responsivity. Nat. Photonics.

[2] Marpaung D., Yao J., Capmany J. (2019). Integrated microwave photonics. Nat. Photonics.

[3] Atabaki A., Moazeni S., Pavanello F., Gevorgyan H., Notaros J., Alloatti L., Wade M.T., Sun C., Kruger S.A., Meng H. (2018). Integrating photonics with silicon nanoelectronics for the next generation of systems on a chip. Nature.

[4] Miritello M., Lo Savio R., Iacona F., Franzo G., Irrera A., Piro A.M., Bongiorno C., Priolo F. (2007). Efficient luminescence and energy transfer in erbium silicate thin films. Adv. Mater..

[5] Muravyev S.V., Anashkina E.A., Andrianov A.V., Dorofeev V., Motorin S.E., Koptev M.Y. (2018). Dual-band Tm^3+^-doped tellurite fiber amplifier and laser at 1.9 μm and 2.3 μm. Sci. Rep..

[6] Nilsson J., Payne D.N. (2011). High-Power Fiber Lasers. Science.

[7] Kim J.H., Holloway P.H. (2005). Near-infrared-electroluminescent light-emitting planar optical sources based on gallium nitride doped with rare earths. Adv. Mater..

[8] Irrera A., Franzo G., Iacona F., Canino A., Di Stefano G., Sanfilippo D., Piana A., Fallica P.G., Priolo F. (2007). Light emitting devices based on silicon nanostructures. Physica E.

[9] Kenyon A.J. (2002). Recent developments in rare-earth doped materials for optoelectronics. Prog. Quantum Electron..

[10] Gu L.L., Xiong Z.H., Chen G., Xiao Z.S., Gong D.W., Hou X.Y., Wang X. (2001). Luminescent erbium-doped porous silicon bilayer structures. Adv. Mater..

[11] Eames C., Probert M.I.J., Tear S.P. (2010). The structure and growth direction of rare earth silicide nanowires on Si(100). Appl. Phys. Lett..

[12] Tatebayashi J., Yoshii G., Nakajima T., Kamei H., Takatsu J., Lebrun D.M., Fujiwaraa Y. (2018). Control of the energy transfer between Tm^3+^ and Yb^3+^ ions in Tm,Yb-codoped ZnO grown by sputtering-assisted metalorganic chemical vapor deposition. J. Appl. Phys..

[13] Yang Y., Li Y.P., Wang C.X., Zhu C., Lv C.Y., Ma X.Y., Yang D.R. (2014). Rare earth doped ZnO films: A material platform to realize multicolor and near infrared electroluminescence. Adv. Opt. Mater..

[14] Zhu C., Lv C.Y., Jiang M.M., Zhou J.W., Li D.S., Ma X.Y., Yang D.R. (2016). Green electroluminescence from Tb_4_O_7_ films on silicon: Impact excitation of Tb^3+^ ions by hot carriers. Appl. Phys. Lett..

[15] Jambois O., Berencen Y., Hijazi K., Wojdak M., Kenyon A.J., Gourbilleau F., Rizk R., Garrido1 B. (2009). Current transport and electroluminescence mechanisms in thin SiO_2_ films containing Si nanocluster-sensitized erbium ions. J. Appl. Phys..

[16] Rebohle L., Braun M., Wutzler R., Liu B., Sun J.M., Helm M., Skorupa W. (2014). Strong electroluminescence from SiO_2_-Tb_2_O_3_-Al_2_O_3_ mixed layers fabricated by atomic layer deposition. Appl. Phys. Lett..

[17] Sun J.M., Prucnal S., Skorupa W., Dekorsy T., Müchlich A., Helm M., Rebohle L., Gebel T. (2006). Electroluminescence properties of the Gd^3+^ ultraviolet luminescent centers in SiO_2_ gate oxide layers. J. Appl. Phys..

[18] Ouyang Z., Yang Y., Sun J. (2018). Near-infrared electroluminescence from atomic layer doped Al_2_O_3_:Yb nanolaminate films on silicon. Scr. Mater..

[19] Yang Y., Li N., Sun J.M. (2018). Intense electroluminescence from Al_2_O_3_/Tb_2_O_3_ nanolaminate films fabricated by atomic layer deposition on silicon. Opt. Express.

[20] Chen Z., Kang S., Zhang H., Wang T., Lv S., Chen Q., Dong G., Qiu J. (2017). Controllable optical modulation of blue/green up-conversion fluorescence from Tm^3+^ (Er^3+^) single-doped glass ceramics upon two-step excitation of two-wavelengths. Sci. Rep..

[21] Lee Y.W., Chien H.W., Cho C.H., Chen J.Z., Chang J.S., Jiang S. (2013). Heavily Tm^3+^-Doped Silicate Fiber for High-Gain Fiber Amplifiers. Fibers.

[22] Lee D.S., Steckl A.J. (2003). Selective enhancement of blue electroluminescence from GaN:Tm. Appl. Phys. Lett..

[23] Guereñu A.L., Bastian P., Wessig P., John L., Kumke M.U. (2019). Energy Transfer between Tm-doped upconverting nanoparticles and a small organic dye with large stokes shift. Biosensors.

[24] Yamada Y., Kanemitsu Y. (2011). Blue light emission from strongly photoexcited and electron-doped SrTiO_3_. J. Appl. Phys..

[25] Chawla V., Ruoho M., Weber M., Chaaya A.A., Taylor A.A., Charmette C., Miele P., Bechelany M., Michler J., Utke I. (2019). Fracture mechanics and oxygen gas barrier properties of Al_2_O_3_/ZnO nanolaminates on PET deposited by atomic layer deposition. Nanomaterials.

[26] Bouriaux L.F., Rosamond M.C., Williams D.A., Davies A.G., Wälti C. (2017). Field-enhanced direct tunneling in ultrathin atomic-layer-deposition-grown Au-Al_2_O_3_-Cr metal-insulator-metal structures. Phys. Rev. B.

[27] Zhang F., Sun G., Zheng L., Liu S., Liu B., Dong L., Wang L., Zhao W., Liu X., Yan G. (2013). Interfacial study and energy-band alignment of annealed Al_2_O_3_ films prepared by atomic layer deposition on 4H-SiC. J. Appl. Phys..

[28] Elam J.W., Routkevitch D., Mardilovich P.P., George S.M. (2003). Conformal coating on ultrahigh-aspect-ratio nanopores of anodic alumina by atomic layer deposition. Chem. Mater..

[29] Bosund M., Mizohata K., Hakkarainen T., Putkonen M., Söderlund M., Honkanen S., Lipsanen H. (2009). Atomic layer deposition of ytterbium oxide using β-diketonate and ozone precursors. Appl. Surf. Sci..

[30] Desirena H., De la Rosa E., Diaz-Torres L.A., Kumar G.A. (2006). Concentration effect of Er^3+^ ion on the spectroscopic properties of Er^3+^ and Yb^3+^/Er^3+^ co-doped phosphate glasses. Opt. Mater..

[31] Goldner P., Pellé F. (1999). Size dependence of the luminescence spectra and dynamics of Eu^3+^: Y_2_O_3_ nanocrystals. J. Lumin..

[32] Sun J.M., Prucnal S., Skorupa W., Helm M., Rebohle L., Gebel T. (2006). Increase of blue electroluminescence from Ce-doped SiO_2_ layers through sensitization by Gd^3+^ ions. Appl. Phys. Lett..

[33] Ouyang Z., Yang Y., Sun J.M. (2018). Electroluminescent Yb_2_O_3_:Er and Yb_2_Si_2_O_7_:Er nanolaminate films fabricated by atomic layer deposition on silicon. Opt. Mater..

